# Supplementary material for: Substrate rugosity and temperature matters: patterns of benthic diversity at tropical intertidal reefs in the SW Atlantic
Source: PeerJ. 2020 Mar 20;8:e8289. doi: 10.7717/peerj.8289 (PMC7087490; doi:10.7717/peerj.8289)
Supplement: Supplementary file 1 [file peerj-08-8289-s001.odt]

|  | Coqueiral | Gramuté | E. das Garças | Costa Bela |
| --- | --- | --- | --- | --- |
|  | Lat. 19° 56' 8.88" S | Lat. 19° 57' 58.68" S | Lat. 20° 2' 12.84" S | Lat. 20° 6' 20.52" S |
|  | Long. 40° 7' 37.2" W | Long. 40° 8' 2.4" W | Long. 40° 9' 50.4" W | Long. 40° 7' 40.8" W |
| Dec | 18/12/17 | 18/12/17 | 16/12/17 | 15/12/17 |
| Jan | - | 16/01/18 | 16/01/18 | 15/01/18 |
| Feb | 16/02/18 | 16/02/18 | 16/02/18 | 16/02/18 |
| Mar | 17/03/18 | 17/03/18 | 17/03/18 | 17/03/18 |
| Apr | 17/04/18 | 17/04/18 | 17/04/18 | 17/04/18 |
| May | 16/05/18 | 16/05/18 | 23/05/18 | 23/05/18 |
| Jun | 28/06/18 | 14/06/18 | 28/06/18 | 29/06/18 |
| Jul | 27/07/18 | 27/07/18 | 26/07/18 | 26/07/18 |
| Aug | 29/08/18 | 28/08/18 | 27/08/18 | 27/08/18 |
