# Supplementary material for: Substrate rugosity and temperature matters: patterns of benthic diversity at tropical intertidal reefs in the SW Atlantic
Source: PeerJ. 2020 Mar 20;8:e8289. doi: 10.7717/peerj.8289 (PMC7087490; doi:10.7717/peerj.8289)
Supplement: Supplementary file 2 [file peerj-08-8289-s002.odt]

|  | df | t | p | r |
| --- | --- | --- | --- | --- |
| AirT | 7 | -1.44 | 0.1918 | -0.48 |
| SST | 7 | -0.98 | 0.3567 | -0.35 |
| P | 7 | -3.39 | 0.0116 | -0.78 |
| Salinity | 7 | 0.53 | 0.6080 | 0.19 |
| SWH | 7 | 0.48 | 0.6477 | 0.18 |
| Cha | 7 | -0.27 | 0.7941 | -0.10 |
|  |  |  |  |  |
| P (autocorrelation removed) | 6 | 1.04 | 0.3383 | 0.39 |
