# Supplementary material for: Substrate rugosity and temperature matters: patterns of benthic diversity at tropical intertidal reefs in the SW Atlantic
Source: PeerJ. 2020 Mar 20;8:e8289. doi: 10.7717/peerj.8289 (PMC7087490; doi:10.7717/peerj.8289)
Supplement: Supplementary file 3 [file peerj-08-8289-s003.odt]

|  | t | p | r |
| --- | --- | --- | --- |
| AirT vs. SST | 6.21 | 0.0004 | 0.92 |
| AirT vs. Precipitation | 2.48 | 0.0416 | 0.68 |
| AirT vs. Salinity | -1.79 | 0.1163 | -0.56 |
| AirT vs. SWH | -2.61 | 0.0344 | -0.70 |
| AirT vs. Chla | -1.52 | 0.1719 | -0.49 |
| SST vs. Precipitation | 1.70 | 0.1325 | 0.54 |
| SST vs. Salinity | -1.22 | 0.2594 | -0.42 |
| SST vs. SWH | -2.17 | 0.0661 | -0.63 |
| SST vs. Chla | -2.67 | 0.0316 | -0.71 |
| Precipitation vs. Salinity | -1.98 | 0.0877 | -0.59 |
| Precipitation vs. SWH | -1.33 | 0.2237 | -0.45 |
| Precipitation vs. Chla | -0.77 | 0.4616 | -0.28 |
| Salinity vs. SWH | 1.67 | 0.1434 | 0.52 |
| Salinity vs. Chla | 0.35 | 0.7324 | 0.13 |
| SWH vs. Chla | 1.01 | 0.3447 | 0.35 |
