# Supplementary material for: Substrate rugosity and temperature matters: patterns of benthic diversity at tropical intertidal reefs in the SW Atlantic
Source: PeerJ. 2020 Mar 20;8:e8289. doi: 10.7717/peerj.8289 (PMC7087490; doi:10.7717/peerj.8289)
Supplement: Supplementary file 4 [file peerj-08-8289-s004.odt]

| Month | AirT | SST | SWH | Chla |
| --- | --- | --- | --- | --- |
| Dec-Jan | < 0.001 | < 0.001 | < 0.001 | 1.000 |
| Dec-Feb | < 0.001 | < 0.001 | < 0.001 | 0.412 |
| Dec-Mar | < 0.001 | < 0.001 | < 0.001 | 0.999 |
| Dec-Apr | < 0.001 | < 0.001 | < 0.001 | 0.015 |
| Dec-May | < 0.001 | < 0.001 | < 0.001 | 0.999 |
| Dec-Jun | 0.970 | 0.999 | < 0.001 | 0.766 |
| Dec-Jul | 0.970 | 0.262 | < 0.001 | 0.279 |
| Dec-Aug | < 0.001 | < 0.001 | < 0.001 | 0.998 |
| Jan-Feb | 1.00 | 0.060 | 0.079 | 0.352 |
| Jan-Mar | 0.407 | 0.001 | < 0.001 | 0.997 |
| Jan-Apr | 0.004 | < 0.001 | 0.229 | 0.011 |
| Jan-May | 0.056 | 0.998 | < 0.001 | 0.999 |
| Jan-Jun | < 0.001 | < 0.001 | < 0.001 | 0.819 |
| Jan-Jul | < 0.001 | < 0.001 | < 0.001 | 0.332 |
| Jan-Aug | < 0.001 | < 0.001 | < 0.001 | 0.999 |
| Feb-Mar | 0.053 | 0.961 | < 0.001 | 0.833 |
| Feb-Apr | 0.995 | < 0.001 | < 0.001 | 0.915 |
| Feb-May | 0.420 | 0.312 | < 0.001 | 0.603 |
| Feb-Jun | < 0.001 | < 0.001 | < 0.001 | 0.005 |
| Feb-Jul | < 0.001 | < 0.001 | < 0.001 | < 0.001 |
| Feb-Aug | < 0.001 | < 0.001 | < 0.001 | 0.100 |
| Mar-Apr | 0.003 | < 0.001 | < 0.001 | 0.101 |
| Mar-May | < 0.001 | 0.014 | < 0.001 | 0.999 |
| Mar-Jun | < 0.001 | < 0.001 | < 0.001 | 0.337 |
| Mar-Jul | < 0.001 | < 0.001 | < 0.001 | 0.061 |
| Mar-Aug | < 0.001 | < 0.001 | < 0.001 | 0.914 |
| Apr-May | 0.919 | < 0.001 | < 0.001 | 0.036 |
| Apr-Jun | < 0.001 | < 0.001 | < 0.001 | < 0.001 |
| Apr-Jul | < 0.001 | < 0.001 | < 0.001 | < 0.001 |
| Apr-Aug | < 0.001 | < 0.001 | < 0.001 | 0.001 |
| May-Jun | < 0.001 | < 0.001 | 0.991 | 0.581 |
| May-Jul | < 0.001 | < 0.001 | < 0.001 | 0.155 |
| May-Aug | < 0.001 | < 0.001 | < 0.001 | 0.987 |
| Jun-Jul | 1.000 | 0.440 | 0.062 | 0.997 |
| Jun-Aug | 0.002 | < 0.001 | < 0.001 | 0.987 |
| Jul-Aug | 0.002 | < 0.001 | 0.494 | 0.724 |
