# Supplementary material for: Substrate rugosity and temperature matters: patterns of benthic diversity at tropical intertidal reefs in the SW Atlantic
Source: PeerJ. 2020 Mar 20;8:e8289. doi: 10.7717/peerj.8289 (PMC7087490; doi:10.7717/peerj.8289)
Supplement: Supplementary file 5 [file peerj-08-8289-s005.odt]

| Site | Chla | Rugosity |
| --- | --- | --- |
| Coqueiral-Gramuté | 0.057 | 0.147 |
| Coqueiral-E.das Garças | 0.449 | 0.012 |
| Coqueiral-Costa Bela | 0.158 | 0.034 |
| Gramuté-E.das Garças | 0.711 | < 0.001 |
| Gramuté-Costa Bela | 0.969 | < 0.001 |
| E. das Garças-Costa Bela | 0.927 | 0.961 |
