# Supplementary material for: Substrate rugosity and temperature matters: patterns of benthic diversity at tropical intertidal reefs in the SW Atlantic
Source: PeerJ. 2020 Mar 20;8:e8289. doi: 10.7717/peerj.8289 (PMC7087490; doi:10.7717/peerj.8289)
Supplement: Supplementary file 6 [file peerj-08-8289-s006.odt]

| Taxonomic group and HTC | Taxonomic group and HTC | Taxonomic group and HTC |
| --- | --- | --- |
| Invertebrates | Macroalgae | Macroalgae |
| Cnidaria | Rhodophyta | Chlorophyta |
| Anemonae | Abyla sp. | Anadyomene stellata |
| Bunodosoma caissarum | Arthrocardia | Caulerpa cupressoides |
| Actinostella flosculifera | Asparagopsis taxiformis | Caulerpa mexicana |
| Corals | Botryocladia occidentalis | Caulerpa cupressoides |
| Colpophyllia natans | Chondracanthus teedei | Caulerpa racemosa |
| Favia fragum | Corallina officinalis | Caulerpa sertularioides |
| Orbicella annularis | Corallina panizzoi | Chaetomorpha spiralis |
| Palythoa sp. | Cryptonemia seminervis | Willeella ordinata |
| Palythoa tuberculosa | Dichotomaria marginata | Cladophora prolifera |
| Siderastrea radians | Gelidiella acerosa | Cladophora vagabunda |
| Siderastrea siderea | Gelidium floridanum | Dictyosphaeria versluysii |
| Solenastrea bournoni | Gracilaria cervicornis | Halimeda cuneata |
| Zoanthus sociatus | Gracilaria domingensis | Udotea flabellum |
|  | Gymnogongrus griffithsiae | Ulva sp. |
| Bivalvia | Hypnea musciformis | Valonia macrophysa |
| Mytilaster solisianus | Jania adhaerens |  |
|  | Jania rubens | Phaeophyta |
| Crustacea | Liagora sp. | Canistrocarpus cervicornis |
| Cirripedia | Lithophyllum sp. | Colpomenia sinuosa |
| Chthamalus bisinuatus | Octhodes secundiramea | Dictyopteris delicatula |
| Anomura | Plocamium brasiliensis | Dictyopteris plagiogramma |
|  | Pterocladiella bartlettii | Dictyota ciliolata |
| Echinodermata | Pterocladiella capillacea | Dictyota crenulata |
| Asteroidea | Spyridia filamentosa | Dictyota menstrualis |
| Echinaster echinophorus | Tricleocarpa fragilis | Dictyota mertensii |
| Echinoidea | Osmundaria obtusiloba | Ectocarpales sp. |
| Echinometra lucunter |  | Hydroclathrus sp. |
|  |  | Lobophora variegata |
| Gastropoda |  | Padina gymnosperma |
| Tegula virudula |  | Sargassum sp. |
| Aplysia dactylomela |  | Sphacelaria sp. |
| Bursatella leachii |  | Spatoglossum schroederi |
|  |  | Stypopodium sp. |
| Porifera |  | Zonaria tournefortii |
