# Supplementary material for: Substrate rugosity and temperature matters: patterns of benthic diversity at tropical intertidal reefs in the SW Atlantic
Source: PeerJ. 2020 Mar 20;8:e8289. doi: 10.7717/peerj.8289 (PMC7087490; doi:10.7717/peerj.8289)
Supplement: Supplementary file 7 [file peerj-08-8289-s007.odt]

| Site | % Cover | Diversity | Richness |
| --- | --- | --- | --- |
| Coqueiral-Gramuté | 0.006 | 0.342 | 1.000 |
| Coqueiral-E.das Garças | 0.006 | 0.012 | 0.114 |
| Coqueiral-Costa Bela | 0.006 | 0.042 | 0.384 |
| Gramuté-E.das Garças | 0.006 | 0.798 | 0.954 |
| Gramuté-Costa Bela | 0.006 | 1.000 | 1.000 |
| E. das Garças-Costa Bela | 0.006 | 1.000 | 1.000 |
