# Supplementary material for: Substrate rugosity and temperature matters: patterns of benthic diversity at tropical intertidal reefs in the SW Atlantic
Source: PeerJ. 2020 Mar 20;8:e8289. doi: 10.7717/peerj.8289 (PMC7087490; doi:10.7717/peerj.8289)
Supplement: Supplementary file 8 [file peerj-08-8289-s008.odt]

| Month | Cover | Diversity | Richness |
| --- | --- | --- | --- |
| Dec-Feb | 0.756 | 1.000 | 1.000 |
| Dec-Mar | 0.056 | 0.140 | 0.028 |
| Dec-Apr | 0.028 | 0.028 | 0.028 |
| Dec-May | 0.028 | 1.000 | 1.000 |
| Dec-Jun | 1.000 | 1.000 | 1.000 |
| Dec-Jul | 0.168 | 0.448 | 0.028 |
| Dec-Aug | 1.000 | 1.000 | 1.000 |
| Feb-Mar | 0.168 | 1.000 | 0.028 |
| Feb-Apr | 0.056 | 0.196 | 0.028 |
| Feb-May | 0.028 | 1.000 | 1.000 |
| Feb-Jun | 1.000 | 1.000 | 1.000 |
| Feb-Jul | 1.000 | 1.000 | 0.280 |
| Feb-Aug | 1.000 | 1.000 | 1.000 |
| Mar-Apr | 0.252 | 1.000 | 1.000 |
| Mar-May | 0.028 | 0.336 | 0.028 |
| Mar-Jun | 0.364 | 0.364 | 0.028 |
| Mar-Jul | 1.000 | 1.000 | 0.224 |
| Mar-Aug | 0.028 | 1.000 | 0.028 |
| Apr-May | 1.000 | 0.056 | 0.056 |
| Apr-Jun | 0.028 | 0.028 | 0.028 |
| Apr-Jul | 0.224 | 1.000 | 1.000 |
| Apr-Aug | 0.028 | 0.560 | 0.028 |
| May-Jun | 0.028 | 1.000 | 1.000 |
| May-Jul | 0.028 | 0.840 | 0.084 |
| May-Aug | 0.028 | 1.000 | 1.000 |
| Jun-Jul | 1.000 | 0.336 | 0.224 |
| Jun-Aug | 1.000 | 1.000 | 1.000 |
| Jul-Aug | 0.140 | 1.000 | 0.336 |
