# Supplementary material for: Substrate rugosity and temperature matters: patterns of benthic diversity at tropical intertidal reefs in the SW Atlantic
Source: PeerJ. 2020 Mar 20;8:e8289. doi: 10.7717/peerj.8289 (PMC7087490; doi:10.7717/peerj.8289)
Supplement: Supplementary file 9 [file peerj-08-8289-s009.odt]

|  | Coqueiral | Gramuté | E. das Garças | Costa Bela |
| --- | --- | --- | --- | --- |
| Month | C | D | R | C | D | R | C | D | R | C | D |
| Dec-Feb | 0.924 | 0.672 | 0.143 | 1.000 | 1.000 | 0.790 | 1.000 | 1.000 | 0.230 | 0.840 | 0.868 |
| Dec-Mar | 0.728 | 1.000 | 0.031 | 1.000 | 1.000 | 0.027 | 0.756 | 0.924 | 0.026 | 1.000 | 1.000 |
| Dec-Apr | 0.896 | 1.000 | 0.350 | 0.728 | 0.812 | 0.035 | 0.532 | 0.700 | 0.033 | 0.868 | 1.000 |
| Dec-May | 0.756 | 1.000 | 0.064 | 0.616 | 1.000 | 0.619 | 0.868 | 1.000 | 0.136 | 0.924 | 0.700 |
| Dec-Jun | 0.812 | 1.000 | 0.446 | 0.868 | 1.000 | 0.264 | 0.952 | 1.000 | 0.050 | 1.000 | 0.840 |
| Dec-Jul | 0.700 | 1.000 | 0.030 | 0.896 | 1.000 | 0.096 | 1.000 | 1.000 | 0.028 | 0.532 | 0.784 |
| Dec-Aug | 0.812 | 1.000 | 0.030 | 0.952 | 1.000 | 0.357 | 1.000 | 0.812 | 0.054 | 1.000 | 1.000 |
| Feb-Mar | 0.840 | 0.924 | 0.028 | 0.756 | 1.000 | 0.059 | 0.728 | 1.000 | 0.028 | 1.000 | 1.000 |
| Feb-Apr | 1.000 | 1.000 | 0.128 | 0.588 | 0.868 | 0.025 | 0.700 | 1.000 | 0.112 | 0.532 | 1.000 |
| Feb-May | 0.784 | 1.000 | 0.023 | 0.924 | 1.000 | 0.850 | 0.896 | 1.000 | 0.823 | 0.672 | 0.476 |
| Feb-Jun | 0.924 | 1.000 | 0.139 | 0.672 | 1.000 | 0.307 | 0.840 | 1.000 | 0.922 | 0.756 | 0.896 |
| Feb-Jul | 0.700 | 0.700 | 0.021 | 0.784 | 1.000 | 0.362 | 0.924 | 1.000 | 0.914 | 0.448 | 1.000 |
| Feb-Aug | 0.840 | 0.868 | 0.030 | 0.868 | 1.000 | 0.299 | 0.840 | 1.000 | 0.594 | 0.644 | 1.000 |
| Mar-Apr | 1.000 | 1.000 | 0.227 | 0.896 | 1.000 | 0.147 | 0.784 | 1.000 | 0.226 | 0.896 | 1.000 |
| Mar-May | 0.896 | 1.000 | 0.618 | 0.980 | 1.000 | 0.059 | 0.924 | 0.728 | 0.039 | 1.000 | 0.756 |
| Mar-Jun | 1.000 | 1.000 | 0.141 | 0.924 | 1.000 | 0.175 | 0.700 | 0.700 | 0.029 | 0.896 | 0.644 |
| Mar-Jul | 0.896 | 1.000 | 0.644 | 0.420 | 1.000 | 0.055 | 0.756 | 0.672 | 0.026 | 0.728 | 1.000 |
| Mar-Aug | 0.812 | 1.000 | 0.862 | 0.756 | 1.000 | 0.028 | 1.000 | 1.000 | 0.046 | 0.896 | 1.000 |
| Apr-May | 0.700 | 1.000 | 0.492 | 0.644 | 1.000 | 0.031 | 0.756 | 0.868 | 0.029 | 0.980 | 0.476 |
| Apr-Jun | 0.756 | 1.000 | 0.788 | 1.000 | 1.000 | 0.025 | 0.756 | 1.000 | 0.034 | 0.868 | 1.000 |
| Apr-Jul | 1.000 | 1.000 | 0.158 | 0.924 | 0.952 | 0.024 | 0.868 | 1.000 | 0.033 | 0.952 | 1.000 |
| Apr-Aug | 0.672 | 1.000 | 0.478 | 0.700 | 0.980 | 0.031 | 1.000 | 1.000 | 0.128 | 0.840 | 1.000 |
| May-Jun | 0.532 | 1.000 | 0.160 | 0.952 | 1.000 | 0.488 | 0.924 | 1.000 | 0.223 | 1.000 | 1.000 |
| May-Jul | 0.784 | 1.000 | 0.348 | 0.924 | 1.000 | 0.596 | 1.000 | 1.000 | 0.164 | 0.840 | 0.840 |
| May-Aug | 0.784 | 1.000 | 0.954 | 0.896 | 1.000 | 0.286 | 0.924 | 1.000 | 0.246 | 0.812 | 0.784 |
| Jun-Jul | 0.700 | 1.000 | 0.050 | 0.588 | 1.000 | 0.882 | 1.000 | 1.000 | 1.000 | 0.728 | 0.868 |
| Jun-Aug | 1.000 | 1.000 | 0.260 | 0.812 | 1.000 | 0.079 | 0.784 | 1.000 | 0.869 | 0.756 | 0.672 |
| Jul-Aug | 0.756 | 1.000 | 0.506 | 0.672 | 1.000 | 0.022 | 0.700 | 1.000 | 0.883 | 0.980 | 0.756 |
