# Supplementary material for: Substrate rugosity and temperature matters: patterns of benthic diversity at tropical intertidal reefs in the SW Atlantic
Source: PeerJ. 2020 Mar 20;8:e8289. doi: 10.7717/peerj.8289 (PMC7087490; doi:10.7717/peerj.8289)
Supplement: Supplementary file 10 [file peerj-08-8289-s010.odt]

|  | Regional | Coqueiral | Gramuté | Enseada | Costa Bela |
| --- | --- | --- | --- | --- | --- |
| H | H = -0.05 + 0.03SST | H = 1.94 - 0.04SST | H = -0.95 + 0.07SST | H = -0.19 + 0.04SST | H = -1.05 + 0.07SST |
| R | R = -1.75 + 0.19SST | R = 5.22 - 0.07SST | R = -5.67 + 0.36SST | R = -2.15 + 0.20SST | R = -4.76 + 0.31SST |
| % Cover |  |  |  |  |  |
| Macroalgae | C = -44.36 + 2.74SST | C = 29.87 + 0.50SST | C = -16.71 + 1.63SST | C = -59.54 + 2.77SST | C = -159.31 + 7.29SST |
| Anthozoa | C = 72.56 - 2.24SST | C = 1.68 - 0.06SST | C = 6.25 + 0.25SST | C = 109.41 - 3.41SST | C = 190.61 - 6.50SST |
| Turf | C = -13.36 + 1.34SST | C = -105.14 + 5.28SST | C = 35.76 - 0.64SST | C = -4.77 + 1.59SST | C = -4.59 + 0.21SST |
| Other | C = -46.43 + 2.05SST | C = -42.84 + 1.83SST | C = 5.52 - 0.12SST | C = -8.73 + 0.40SST | C = -139.50 + 6.10SST |
